# Age/BMI is a Stronger Predictor of Death in COVID-19 Patients than Age Alone: A Pilot Study

**DOI:** 10.1007/s44197-022-00075-z

**Published:** 2022-11-10

**Authors:** Wala Al Balwi, Maha Al Turki, Ziad A. Memish, Hana M. A. Fakhoury, Mohammed Al Balwi, Ali H. Hajeer

**Affiliations:** 1 College of Applied Medical Sciences, King Saud Bin Abdulaziz University for Health Sciences, Riyadh, Saudi Arabia; 2 Research & Innovation Centre, King Saud Medical City, Ministry of Health Riyadh, Riyadh, Saudi Arabia; 3 Department of Biochemistry and Molecular Medicine, College of Medicine, Alfaisal University, Riyadh, Saudi Arabia; 4 Department of Pathology and Laboratory Medicine, College of Medicine, KSAU-HS, Ministry of National Guard Health Affairs, Riyadh, Saudi Arabia

**Keywords:** COVID-19, SARS-CoV-2, Age/BMI score, Saudi

## Abstract

The objective of this study was to investigate the effect of age and BMI on the risk of death in patients with coronavirus disease 2019 (COVID-19). A cohort of 206 Saudi COVID-19 patients was included in this study. Data on age, BMI, hospitalization, comorbidities, and death were collected and analyzed. Descriptive, univariate, and multivariate logistic regression analyses were carried out. Out of the 206 studied patients, 28 died. Hypertension, cardiac disease, and hospital admission were predictors of death in univariate and multivariate logistic regression analysis. Moreover, age was a significant predictor of death, while increased BMI seemed to be protective at an older age. Therefore, a new score was suggested taking into consideration both factors, namely age/BMI score. Although older age was associated with death in univariate (OR, 1.09 [95% CI 1.05–1.12], *p* < 0.001) and multivariate analysis (OR, 1.05 [95% CI 1.02–1.09], *p* = 0.004), a higher age/BMI score was a stronger predictor of death than age alone, in both univariate (OR 4.42 [95% CI 2.50–7.80], *p* < 0.001) and multivariate analysis (OR 3.11 [95% CI 1.66–5.82], *p* < 0.001). Several factors appear to contribute to the risk of COVID-19 death. Interestingly, our new age/BMI score seems to carry a higher risk of death than age alone. This new score will be designated as the Hajeer score. Since this is a small cohort study, we recommend investigating this score in a larger cohort.

## Introduction

Coronavirus disease 2019 (COVID-19) is caused by a novel coronavirus known as severe acute respiratory syndrome-Coronavirus (SARS-CoV-2). It was first identified in Wuhan City, China at the end of 2019, later the disease spread to the rest of the world [1]. In March 2020, the World Health Organization (WHO) announced COVID-19 as a global pandemic [2] accounting for more than six million deaths worldwide as of July 2022.

Clinically, COVID-19 symptoms vary from asymptomatic to severe disease [3]. Severe cases of COVID-19 lead to pneumonia and respiratory failure with the need for hospitalization and critical care [4]. It has been reported that severe COVID-19 cases may lead to acute respiratory distress syndrome, septic shock, acute renal failure and multisystem organ failure [5].

Several factors were found to predispose to risk of death in COVID-19 patients, including older age and the presence of comorbidities such as hypertension, diabetes, obesity and smoking [6, 7]. As such, the characteristics of the clinical presentations of COVID-19 may vary according to the demographics and epidemiological profiles of each country.

The analysis of data from patients with COVID-19 hospitalized at 88 US hospitals enrolled in the American Heart Association's COVID-19 Cardiovascular Disease Registry [8], demonstrated that BMI ≥ 40 was a strong risk factor for death in adults ≤ 50 years (OR, 1.64 [95% CI 1.23–2.21]). However, this risk was intermediate in 51–70 years old (OR, 1.40 [95% CI 1.10–1.80]), and non-significant in > 70 years old (OR, 1.28 [95% CI 0.83–1.95].

Furthermore, a study in South Korea by Cho et al. [9] found that being underweight was a risk for death while being overweight was associated with lower risks of disease severity and mortality in older patients with COVID-19.

Here, we investigated the risk for death in a cohort of COVID-19 patients. We found that age was associated with death while increasing BMI appeared to be protective against death. Therefore, we devised a new score based on our own findings and others [8, 9], that is age/BMI score. The objective of this study was to investigate the risk of death in COVID-19 patients using known comorbidities and our new age/BMI score.

## Materials and Methods

### Data Collection and Patient Phenotype

This is a retrospective cohort study comprising a convenience sample of randomly selected 206 COVID-19 patients from King Abdulaziz Medical Center, Ministry of National Guard Health Affairs, Riyadh, Saudi Arabia. All patients tested positive using COVID-19 RT-PCR. A total of 99 patients (48.1%) were hospitalized due to severe illness. The study was approved by the Institutional Review Board (IRB) at King Abdullah International Medical Research Center [SP21R/266/05]. Confirmed COVID-19 cases between April 2020 and August 2021 and aged above 16 years were included. Relevant data on gender, BMI, comorbidities, and hospital admission were retrieved from the electronic patient records.

### Main Outcome Measure

The main outcome measure for this study was in-hospital death following COVID-19 diagnosis.

### Age/BMI

Based on our preliminary finding that age was positively associated with risk of death in our cohort and BMI was negatively associated with death, we coined a new scoring system which is the ratio of age over BMI (age/BMI).

### Statistical Analysis

Patients’ basic descriptive data and clinical presentation, complications, and admission were summarized as frequencies and percentages. Continuous data of the descriptive statistics were presented as mean and standard deviation. Pearson Chi-square test was used to analyze categorical variables and Student’s *t* test for continuous variables. Univariate analysis (Table 2) was performed against our primary outcome (death), and variables significant at *p* < 0.05 were selected for inclusion in our multivariate logistic regression models. Two multivariate logistic regression models were carried out; one with age, BMI, and comorbidities (Model 1, Table 3); the other with age/BMI score and comorbidities, excluding age and BMI (Model 2, Table 3). Results were presented as odds ratio (OR) and 95% confidence interval (CI). Statistical analysis was performed with STATA version 15.0 software (StataCorp, College Station, TX).

## Results

A total of 99 patients (48.1%) of the studied cohort were hospitalized, of which 27 (13.1%) were non-survivors. The mean age of the total cohort was 55.56 ± 18.41 years, while the non-survivors were significantly older (74.04 ± 14.45) than the survivors (52.65 ± 17.14) (*p* < 0.001). The mean BMI for the total group was 30.65 ± 10.20, with non-survivors having a lower mean BMI (28.53 ± 8.46) than survivors (30.99 ± 10.43) (*p* = 0.557). A total of 46.6% of the group were males, which rose to 75% in the non-survivors (*p* = 0.001). The following comorbidities were more common in non-survivors, namely diabetes (not a statistically significant difference), hypertension (*p* < 0.001), and cardiac disease (*p* = 0.008) (Table 1).

**Table 1:** Patients’ demographic data

Variable	Survivors *N* = 178	Non-survivors *N* = 28	Total *N* = 206	*p* value
Mean age in years ± SD	52.65 ± 17.14	74.04 ± 14.45	55.56 ± 18.41	**< 0.001**
Mean BMI in Kg/m^2^ ± SD	30.99 ± 10.43	28.53 ± 8.46	30.65 ± 10.20	0.557
Mean age/BMI score	1.78 ± 0.66	2.87 ± 1.13	1.93 ± 0.83	**< 0.001**
Male gender N (%)	75 (42.10)	21 (75.00)	96 (46.60)	**0.001**
Diabetes N (%)	84 (47.19)	16 (57.14)	100 (48.54)	0.327
Hypertension N (%)	63 (35.40)	21 (75.00)	84 (40.80)	**< 0.001**
Cardiac disease N (%)	13 (7.30)	8 (28.6)	21 (10.20)	**0.008**
Hospital admission N (%)	72 (40.50)	27 (96.40)	99 (48.10)	**< 0.001**

Bold values denote statistical significance

Univariate analysis (Table 2) demonstrated that the following variables were risk factors for death in our cohort, namely age (OR, 1.09 [95% CI 1.05–1.12], *p* < 0.001), male gender (OR, 4.12 [95% CI 1.67–10.19], *p* = 0.002), hypertension (OR, 5.48 [95% CI 2.10–13.59], *p* < 0.001), cardiac disease (OR, 5.08 [95% CI 1.88–13.74], *p* = 0.001), and hospital admission (OR, 39.75, [95% CI 5.28–299.12], *p* < 0.001). On the other hand, BMI was protective against death. Indeed, our results suggested that the higher the BMI the lower the risk of death (OR, 0.96 [95% CI 0.90–1.02], *p* = 0.184), although this did not reach statistical significance. In multivariate analysis (Table 3 model 1), age, cardiac disease, and hospital admission were still significant.

**Table 2:** Univariate analysis of risk factors for death in COVID-19 patients

Univariate analysis			
Variable	OR	*p*	95% CI
Age	1.09	**< 0.001**	1.05–1.12
BMI	0.96	0.184	0.90–1.02
Age/BMI score	4.42	**< 0.001**	2.50–7.80
Male gender	4.12	**0.002**	1.67–10.19
Hypertension	5.48	**< 0.001**	2.10–13.59
Cardiac disease	5.08	**0.001**	1.88–13.74
Hospital admission	39.75	**< 0.001**	5.28–299.12

Bold values denote statistical significance

**Table 3:** Multivariate analysis of risk factors for death in COVID-19 patients. Model 1 used age, BMI and the significant univariate variables. Model 2 used age/BMI and the significant univariate variables

Variable	Multivariate		
	OR	p	95% CI
Model 1			
Age	1.05	**0.008**	1.01–1.09
BMI	0.96	0.376	0.88–1.05
Male gender	0.74	0.619	0.23–2.41
Hypertension	2.69	0.102	0.82–8.82
Cardiac disease	3.79	**0.038**	1.08–13.34
Hospital admission	24.09	**0.004**	2.84–204.29
Model 2			
Age/BMI score	3.11	**< 0.001**	1.66–5.82
Male gender	1.08	0.902	0.34–3.43
Hypertension	3.93	**0.020**	1.24–12.47
Cardiac disease	3.97	**0.037**	1.09–14.51
Hospital admission	28.94	**0.002**	3.47–248.30

Bold values denote statistical significance

Based on our findings, we devised a new scoring system, namely age/BMI score, and we called it the “Hajeer score”. Results for this score in our cohort ranged between 0.42 and 4.9. We used this new scoring system in univariate and multivariate analyses. Interestingly, univariate analysis (Table 2) showed that age/BMI was a significant risk factor for death in our cohort and carried a higher risk than age alone (OR, 4.42 [95% CI 2.50–7.80], *p* < 0.001). Moreover, when a multivariate analysis of age/BMI and significant univariate variables was conducted, all variables were significant except for male gender (Table 3 model 2).

## Discussion

In this study, we analyzed the risk of death in a cohort of COVID-19 patients. Similar to previously published data, we found that age, male gender, hypertension, cardiac disease, and hospitalization were risk factors for death.

The Hajeer score is our proposed new scoring system which is the ratio of age/BMI. This suggested scoring system is based on our as well as others’ findings [8, 9] that older age is a risk for death while higher BMI was protective in the elderly. Interestingly, the higher the score the higher the risk of death in this cohort of COVID-19 patients. Our findings showed that age carried a 10% risk of death in both univariate and multivariate analysis, while age/BMI carried a 400% risk of death in univariate and 300% risk in multivariate analysis. This suggests that our new score is a much better predictor of death in COVID-19 patients than age alone.

In a meta-analysis, Du et al. [10] found that BMI was associated with COVID-19 severity and mortality. Evidence from this meta-analysis suggested a linear dose–response association between BMI and both COVID-19 severity and mortality. Moreover, obesity (BMI ≥ 30 kg/m^2^) was associated with a significantly increased risk of critical COVID-19 and in-hospital mortality of COVID-19. However, Li et al. [11] found that malnutrition and low BMI are strong predictors of severe outcomes in COVID-19 patients. Evidence suggests that a higher BMI appears to increase the risk of death in younger patients, but protects against death in the elderly [9, 12]. Similarly, a cohort study conducted in Denmark demonstrated that upper and lower respiratory infections were associated with BMI in a U-shaped relationship, suggesting an increased risk in underweight patients [13].

Based on our results and those of similar studies, we propose that a low BMI in the elderly (thus a higher age/BMI score) might reflect malnutrition and frailty. In this respect, our new score might be a better predictor of death in older COVID-19 patients.

Our study is limited by its small sample size, and we recommend further larger studies to investigate our new scoring system and COVID-19 death. In addition, data on vaccination status is lacking since some of the cases were included before the availability of the vaccine. Although sequencing of the SARS-CoV-2 variant was not performed, one could suggest that it was the most common variant present in the middle east during the period of the study, namely the alpha variant.

## Conclusion

Our study concluded that age, male gender, hypertension, cardiac disease, and hospitalization are risk factors for death in our cohort of COVID-19 patients. In addition, our new score, namely age/BMI appeared to carry a higher risk of death than age alone, suggesting that older COVID-19 patients with lower BMI are at greater risk of death. This is a small cohort study, and we recommend investigating our new score in a larger cohort of patients.

## Data Availability

The data of the study, including the code used in the analyses, are available from the corresponding author upon reasonable request.

## Abbreviations

SARS-CoV-2: Severe acute respiratory syndrome coronavirus 2
COVID-19: Coronavirus disease 2019
PCR: Polymerase chain reaction
